# Surgery for stress urinary incontinence in women: A 2006 review

**DOI:** 10.4103/0970-1591.32066

**Published:** 2007

**Authors:** Bertil F. M. Blok, Jacques Corcos

**Affiliations:** Department of Urology, Jewish General Hospital, McGill University, Montreal, Quebec, Canada

**Keywords:** Articifial urinary sphincter, cervicocystopexy, injectables, slings, stress urinary incontinence, tension free vaginal tape, urinary incontinence

## Abstract

The surgical treatment of female stress urinary incontinence is a rapidly changing field. This review discusses recent advances in various injectables, minimally invasive techniques and open procedures. It particularly evaluates data from long-term outcome studies and describes peri- and postoperative complications from several procedures, such as bulking agents, tension-free vaginal tape and its modifications (TOT, TVT-O) as well as open and laparoscopic colposuspension.

Urinary incontinence is a common condition that affects a large part of the female population. One in every nine women will undergo surgery for pelvic floor dysfunction.[1] A recent, large, population-based survey of American women aged 30-90 years reported that the prevalence of urinary incontinence was 45% overall, ranging from 28% among 30- to 39-year-olds to 55% among 80- to 90-year-olds.[2]

Urinary incontinence can originate from the urethra or bladder or from a combination of both. Bladder causes are detrusor hyperactivity or hypoactivity. Detrusor hyperactivity may result in urge incontinence and detrusor hypoactivity may lead to overflow incontinence. Urethral causes of stress incontinence are thought to be due to intrinsic urethral sphincter deficiency with or without urethral hypermobility. The initial management of stress incontinence is usually conservative and consists of behavioral and lifestyle modification programs, like supervised pelvic floor exercises, weight loss, smoking cessation and altered fluid intake. These treatments can give adequate symptom relief, but have to be maintained continuously and do not cure the incontinence. Surgical intervention is considered in women who fail initial conservative management or who want to avoid long-term nonsurgical treatment.

For a long time, we have underscored the existence of a primary sphincteric deficiency in all cases of stress urinary incontinence (SUI). Except for pelvic floor exercises, none of the present treatments addresses this deficiency, being based mainly on restoration of the sling effect (bladder neck suspensions, slings) or increased urethral resistance (bulking agents, artificial sphincters).

There are many operations for correcting SUI, but every procedure is not always successful or without complications. Our review mainly discusses recent advances in this quickly-moving field, but will also refer to less recent procedures that still have their proper indications.

## MINIMAL WORKUP

Before considering surgery, it is essential to identify the roles of the bladder and the sphincter which could be different for each incontinent patient. Furthermore, patient suitability and expectations from the chosen procedure should be assessed beforehand. These will depend on the cause of the incontinence and the balance of risks and benefits to her.

Physical examination, symptoms and quality of life or impact questionnaires as well as voiding diaries are considered the minimum workup for all women. Urodynamic recording, including filling, voiding studies and urethral function assessment, is mandatory only if the patient has had previous pelvic floor surgery, presents overactive bladder symptoms and has a history of neurological disease or any other condition where the diagnosis of SUI is not straightforward. Urodynamic testing will confirm the diagnosis of SUI by ascertaining leak point pressure, will show a possible element of detrusor hyperactivity and exclude voiding difficulties as well as postvoid residual. In case of high-amplitude, uninhibited detrusor contractions, loss of compliance and decreased bladder capacity, surgery is unlikely to improve the incontinence and could even worsen bladder over-activity. Minor detrusor contractions during or at the end of filling are more controversial; they are usually associated with symptoms of mixed incontinence and don't represent at our point of view a contraindication to surgery. However, each case has to be studied cautiously and the patient informed of the risk of persistent over-activity after surgery. Finally, to complete the investigation, a 24-h pad test is recommended to quantify the incontinence.

**Minimum assessment of SUI before surgery**

- Symptoms questionnaire
- Quality of life and/or impact questionnaire
- Physical examination with the stress test
- 24-h pad test

In case of recurrence, mixed incontinence, discrepancy between the clinical findings and complaints of neurogenic incontinence:

- Urodynamic study
- Cystoscopy
- Other specialized tests

## OPERATIONS

A complete historical overview of the many operations that have been designed to counter SUI is beyond the scope of this review. The aim of incontinence surgery is to elevate and support the proximal urethra and bladder neck to rebuild the physiological sling mechanism created by the muscles and ligaments of the pelvic floor.

Prior to invasive surgery, urethral injections with bulking agents can be considered to increase urethral resistance. Mid-urethral tapes, which aim to restore continence with minimal alteration of the pelvic floor anatomy and minimal resistance to urine flow, are more recently-introduced, minimally-invasive techniques or procedures. Sub-mid-urethral slings made from organic or inorganic materials with numerous modifications offer the same benefits with a less invasive technique in most cases than traditional “open” colposuspensions/slings. Although less and less fashionable, Burch colposuspension is still considered the standard to which other procedures must compare. We will discuss these new techniques and their applications.

## INJECTABLE BULKING AGENTS

Various biological and nonbiological products have been developed for periurethral injection. The ideal material has to be nonallergenic, nonimmunogenic, noncarcinogenic, retain its bulking characteristics for a long time and not migrate or degrade. It also needs to be easily injected into the urethra. Currently available bulking agents all have to be injected more than once to achieve a satisfactory increase of urethral resistance. Most commonly used are glutaraldehyde cross-linked bovine collagen (Contigen), silicone particles (Macroplastique), carbon particles (Durasphere) and dextranomer/hyaluronic acid copolymer (Zuidex).

**Figure 1:**
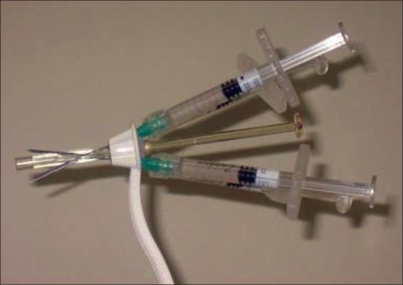
The implacer and syringes of the ZUIDEX system

**Figure 2:**
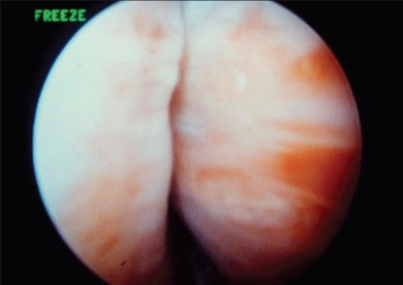
Aspect of a woman's proximal urethra after collagen injections

The urethral injection technique is quite similar for most agents. It can be delivered transurethrally through a cystoscope with a 22-gauge needle. Alternatively, a device placed in the urethra directs injections along a needle track [Figure 1]. Most procedures can be performed on an outpatient basis under local anesthesia. The aim of bulking agent injections is to narrow the bladder neck [Figure 2]. The short-term overall complication rate is low (< 5%) and comprises outflow obstruction, urinary tract infection and hematuria. Sterile abscess formation can occur one year postoperatively in association with irritative voiding symptoms, pelvic pain, urinary incontinence and a tender periurethral mass.[3] Long-term complications are rare, but it has been reported recently that 13% of children receiving glutaraldehyde cross-linked collagen at the bladder neck developed calcifications at the site of prior injections with a mean follow-up of 8.8 years.[4] *De novo* urgency can still occur in up to 13% of patients. Cure rates to the order of 20 to 70% have been reported, but many women will require re-injection within a few years of the initial operation.[5–7]

## TENSION-FREE MIDURETHRAL TAPES (TVT, TOT, TVT-O AND OTHERS)

**Figure 3:**
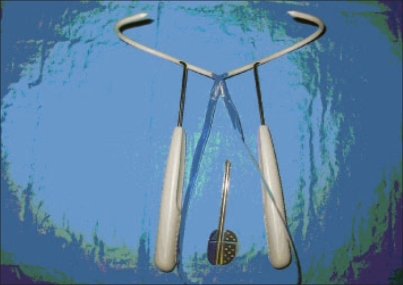
The TVT-O kit

**Figure 4:**
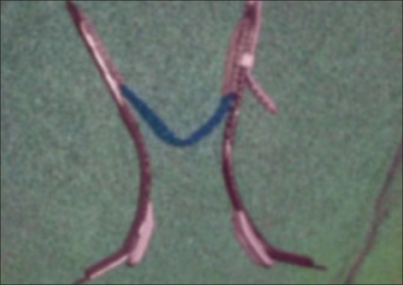
The TVT-Secur system

This technique, introduced by Ulmsten and colleagues,[8] shifted the surgical focus from bladder neck to midurethral support. The goal of the tension-free vaginal tape (TVT) technique is to correct inadequate urethral support. The tape is positioned free of tension under the midurethra without repositioning the bladder neck. The tape itself is a prolene mesh covered with a protective, transparent plastic sleeve and attached to two needles. The sleeve allows easy passage of the mesh through the tissues without unwanted friction and protects the mesh from contamination during insertion. The original procedure has undergone many modifications by surgeons since its introduction, but remains the same in principle. At present, there are essentially three techniques, but numerous tapes are sold by different companies: 1) the original TVT;[8] 2) the outside-in tension-free obturator tape (TOT)[9] and 3) the inside-out tension-free vaginal tape (TVT-O;[10] [Figure 3]). Tapes on the market are based on one of these three principles. They are usually installed under spinal or local anesthesia and antibiotic prophylaxis is given perioperatively. In mid-2006, a completely new concept of suburethral tape was introduced by Johnson and Johnson Women Care. The “TVT-Secur”, a very short tape introduced without a needle in the periurethral space, is intended to decrease the complication rate with the above-mentioned tapes. No clinical results are available for this new tape [Figure 4].

Peroperative complications of midurethral tapes are related to the insertion technique and whether or not the retropubic space is used as an entrance to the urethra. Blind passage of the trocar through the retropubic space in the TVT procedure has been associated with several problems resulting from penetration of the bladder, urethra, bowels, nerves and vessels.[11] Bladder perforation is the most common complication occurring during the TVT procedure, with a reported incidence of 0.8-21%.[11] In the TOT and TVT-O procedures, the tape is placed more horizontally between the two ischiopubic rami without traversing the retropubic space. The risk of bladder and urethral perforation appears to be highly reduced with these newer techniques. Postoperative complications of midurethral tape procedures include voiding disorders, such as urinary retention and *de novo* urgency, similar to problems that occur after conventional pubovaginal sling implantation. Tension-free obturator tape has been shown to be as effective as TVT with less intraoperative and postoperative complications.[12] However, TOT has been linked with higher rates of erosion and infection than TVT-O,[13] probably due, in part, to the difference in mesh.[14]

The objective and subjective cure rates of midurethral tapes are comparable to those of Burch colposuspension. Long-term studies (five-year follow-up) have reported objective and subjective cure rates of 85%.[15] However, as yet, there has been no longer-term follow-up (>10 years); patients should be informed of this if they decide to undergo the procedure.

## OPEN AND LAPAROSCOPIC COLPOSUSPENSION

Open colposuspension was first described by Burch in 1961[16] and its modification by Tanagho[17] is most widely used at present. The procedure is aimed at elevating paraurethral tissues by suturing to the ipsilateral iliopectineal (Cooper) ligaments. This results in increased support of the urethra and bladder neck by attachment to the lateral pelvic wall.

Postoperative complications, such as voiding problems (3-32%), *de novo* urgency (3.4-18%), pelvic organ prolapse and enterocele (3-17%), are relatively common within five years.[18] However, the available literature does not report a higher complication rate than for other surgical techniques to correct SUI.

A recent Cochrane review[19] included 39 trials involving a total of 3,301 women. Long-term follow-up showed an overall cure rate between 68.9-88.0%, with a one-year cure rate of approximately 85-90%. Alcalay *et al*[20] reported that the cure rate remained stable at 69% for 10-12 years, making it one of the most effective long-term treatments of SUI.

A laparoscopic version of colposuspension has been developed. Like many other laparoscopic procedures, it reproduces and replaces the open technique with the aim of achieving shorter hospital stay, less pain and morbidity, with an earlier return to daily activities. The long-term results of laparoscopic colposuspension remain uncertain. A recent Cochrane review concluded that it may be as good as open colposuspension at two years postsurgery,[21] but solid results with follow-up of five years or more are not available. Furthermore, the newer minimally invasive tape procedures offer even greater benefits and probably better objective outcomes in the long-term than the laparoscopic procedure.

## OTHER PROCEDURES

### Traditional pubovaginal sling

Since the introduction of minimally invasive tension-free midurethral sling procedures, there remain only a few indications for combined transabdominal and transvaginal placement of the pubovaginal sling at the bladder neck. It has been suggested that only the autologous fascia sling has good long-term results,[22,23] but no reliable, long-term, prospective trials have judged the success rate of the traditional pubovaginal sling.[24] In view of the longer operation time, surgical morbidity and relatively frequent postoperative voiding dysfunction, the pubovaginal sling procedure should only be considered in selected cases, for example, women with vaginal wall atrophy due to radiotherapy, lack of urethral mobility, mixed urinary incontinence with open bladder neck, concomitant urethral reconstruction or after failed incontinence procedures.

### Artificial urinary sphincter (AUS)

Use of the AUS in women has been reserved for patients with intrinsic sphincter deficiency due to multiple failed anti-incontinence operations or congenital abnormalities. The AUS may be placed transabdominally and/or transvaginally with the sphincter cuff at the proximal urethra near the bladder neck; the device should be deactivated during the last weeks of pregnancy. Although the long-term continence rate with the AUS in women is excellent, it has been shown that after seven years only 37% of the original AUS were still *in situ*.[25] In other women, it was replaced or removed due to erosion, infection (46%) or late mechanical failure (17%).

Recommended algorithm for treatment of SUI in women

1. Changes in lifestyle (weight loss, smoking cessation, regular exercise, etc.)
2. Physiotherapy of the pelvic floor (under adequate supervision)
3. If no associated prolapse and absent or moderate bladder neck mobility (BNM): bulking agents (BA)
4. BA failure, associated prolapse, major BNM: midurethral sling
5. Recurrent SUI: redo midurethral sling or BN fascial sling
6. Major SUI (posttraumatic) or after several treatment failures: artificial sphincter

## SUMMARY

Recent advances in the surgical treatment of SUI indicate that minimally invasive midurethral tape procedures might become the next gold standard in the near future. However, we do not have complete knowledge of the long-term outcome of these newer techniques and patients need to be informed about this lack of information. Furthermore, the near future will also present data from clinical trials of transurethral injections of myoblasts and other differentiated cells into deficient urethral sphincters.[26] It has to be proven whether these molecular biological techniques result in true functional improvement or rather represent a bulking effect which increases urethral resistance in SUI. If the functionality of these implants is proven, it will be the first treatment of SUI that addresses its real pathophysiology of sphincteric deficiency.
